# Reduction of Operating Current by Harnessing the Field‐ and Damping‐Like Torque Ratios in Nonmagnet–Ferromagnet Heterojunctions

**DOI:** 10.1002/smsc.202300224

**Published:** 2023-12-15

**Authors:** Min Hyeok Lee, Seok-Jong Kim, Seok In Yoon, Jeong Kyu Lee, Han Seok Ko, Gyusang Kim, Seokhie Hong, Kyung-Jin Lee, Young Keun Kim

**Affiliations:** ^1^ Department of Materials Science and Engineering Korea University Seoul 02841 Republic of Korea; ^2^ Department of Physics Korea Advanced Institute of Science and Technology (KAIST) Daejeon 34141 Republic of Korea; ^3^ Department of Materials Science and Engineering Korea Advanced Institute of Science and Technology (KAIST) Daejeon 34141 Republic of Korea; ^4^ Institute of Cyber Security & Privacy (ICSP) School of Cybersecurity Korea University Seoul 02841 Republic of Korea

**Keywords:** damping-like torque, field-like torque, heterojunctions, spin–orbit torque, true random number generators

## Abstract

With the growing demand for high‐speed electronic devices with low energy consumption, spin–orbit torque (SOT) has become a significant focus. SOT can switch the magnetization direction in a material system with broken inversion symmetry, such as a normal metal (NM)/ferromagnet (FM) heterojunction. The SOT consists of two mutually orthogonal vector components along with the injected current direction: the transverse damping‐like torque (DLT) and the longitudinal field‐like torque (FLT). Numerous studies have mainly centered on the DLT for the SOT switching mechanism. However, DLT and FLT are essential to enhance SOT efficiency because FLT boosts the magnetization precession motion. Herein, heterojunctions consisting of NM 1 (Ta, W, or Pt)/NM 2 (Nb)/FM (CoFeB) are devised to manipulate the FLT‐to‐DLT ratio (*η*) through the change in Nb thickness. Furthermore, experimental confirmation exists for reducing threshold current as *η* increases. The SOT devices with substantial *η* generate random numbers. The National Institute of Standards and Technology Special Publication 800‐90B test verifies randomness and confirms that the SOT devices are beneficial sources for true random number generators (TRNGs). These findings indicate the crucial role of FLT in the SOT switching process and underscore its significance in developing SOT‐based TRNG devices.

## Graphical abstract

Theoretically, superior field‐like torque (FLT) in spin–orbit torque (SOT) devices can reduce switching energy. The study experimentally explores the FLT‐to‐damping‐like torque ratio in normal metal (NM) 1/NM 2/ferromagnet heterojunctions, revealing switching current density behavior. Furthermore, the random bits generated from high FLT‐SOT devices exhibit high entropy, showcasing the potential as true random number generators.

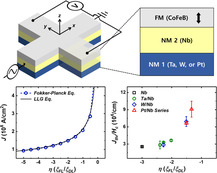

## 1 Introduction

Spin–orbit torque (SOT) is one of the promising candidates for devices that combine the spin of electrons and electronic devices. Many studies have developed materials with a significant SOT efficiency for low switching current density (*J*_sw_) and reduced energy consumption. In‐plane charge currents can generate spin currents in normal metal (NM)/ferromagnet (FM) thin‐film heterojunctions by the spin Hall effect in bulk and the Rashba effect at the interface of NM/FM.^[^ ^1^, ^2^, ^3^, ^4^, ^5^, ^6^, ^7^, ^8^ ^]^ The injected in‐plane charge current along the *x*‐axis induces the *z*‐flow spin current polarized along the *y*‐axis. The spin current can transfer its angular momentum to the FM layer by SOT. The SOT consists of two mutually orthogonal vector components: damping‐like torque (DLT, $\tau_{\text{DLT}}=\hat{m}\times \left(\hat{m}\times \hat{s}\right)$) and field‐like torque (FLT, $\tau_{\text{FLT}}=\hat{m}\times \hat{s}$), where $\hat{m}$ and $\hat{s}$ are unit vectors of magnetization and spin polarization.^[^ ^9^, ^10^, ^11^, ^12^, ^13^, ^14^, ^15^, ^16^, ^17^, ^18^ ^]^

This study explores the potential of SOT devices with perpendicular magnetic anisotropy (PMA) as a foundation for true random number generators (TRNGs) based on stochastic physics. The DLT aligns the magnetization along the *y*‐direction. Upon the cessation of the current supply, the magnetization undergoes random reorientation along the favorable magnetization axis (±*z*) through thermal fluctuations. This stochastic magnetization arrangement facilitates the production of random sequences, as the possibilities of aligning in the +*z* and −*z* orientations are equivalent in a perfect PMA condition. As a result, active exploration of SOT‐based TRNGs, which leverage intrinsic stochastic properties, is underway to replace the complementary metal–oxide–semiconductor (CMOS)‐based RNGs that require postprocessing steps to ensure randomness.^[^ ^19^, ^20^, ^21^, ^22^, ^23^, ^24^ ^]^

Prior SOT studies ignore the influence of FLT on the switching process. Most previous studies have not analyzed the FLT impact, focusing solely on analyzing DLT during switching operations.^[^ ^9^, ^11^, ^12^, ^21^, ^22^, ^23^, ^24^, ^25^, ^26^, ^27^, ^28^ ^]^ Similarly, when we utilize SOT as the underlying basis for generating random numbers, consideration often centers on the effects of DLT rather than FLT. Nevertheless, recent investigations reported that FLT substantially affects the switching functionality within SOT systems. Theoretical conjectures further suppose that FLT is crucial in mitigating the *J*_sw_.^[^ ^29^, ^30^, ^31^, ^32^ ^]^ In this context, the realization of high‐performance SOT‐based TRNGs necessitates the development of predominant material structures influenced by FLT and mandates a systematic investigation into their consequential effects. Moreover, it remains imperative to ascertain the durability of the underlying physical mechanisms that give rise to random number generation.

Here, we theoretically predict the effect of FLT and confirm that with the ascendancy of FLT, the operational threshold current (*J*_thr_) experienced a reduction while maintaining the switching probability of half. *J*_thr_ is the current density that completely rotates the perpendicular magnetization to the in‐plane without an external magnetic field. It is distinct from *J*_sw_, the current density required for deterministic switching. Subsequently, we confirm the theoretical prediction experimentally. We designed the NM 1/NM 2/FM heterojunctions consisting of an NM 1 with Ta, Pt, or W, an NM 2 with Nb, and an FM with CoFeB to facilitate modulating the *η*. We expected to handle the DLT and FLT by varying the NM 2 thickness. We measured the FLT (*ξ*_FL_) and DLT efficiency (*ξ*_DL_) using a harmonic Hall voltage measurement. The experiment results revealed that the parameter *η* exhibited modulation corresponding to the augmentation in the thickness of NM 2. Furthermore, employing micrometer‐scaled SOT devices, we generated random numbers via SOT‐induced switching measurement. We conducted the National Institute of Standards and Technology Special Publication (NIST SP) 800‐90B test and confirmed their performance as a TRNG application.

## 2 Results and Discussion

### 2.1 Role of the FLT Acting on the Magnetization Switching

The magnetization dynamics induced by SOT is described by the following Landau–Lifshitz–Gilbert (LLG) equation:

(1) $$\dot{\hat{m}}\;=-\gamma \hat{m}\;\times H_{eff}+\alpha \hat{m}\;\times \dot{\hat{m}}\;+\gamma J\left(h_{D}\hat{m}\;\times \left(\hat{m}\;\times \hat{y}\right)+h_{F}\hat{m}\;\times \hat{y}\right)$$

where *γ* is the gyromagnetic ratio, $H_{eff}=H_{k}m_{z}\hat{z}$ is the effective field acting on $\hat{m}$, which includes PMA in the magnitude of *H*_*k*_, *α* is the damping constant, *h*_D(F)_ (= ℏ*ξ*_D(F)_/2e*M*_s_ *t*_F_) describes the magnitude of the DLT (FLT), *J* is the magnitude of applied current density, and *M*_s_ and *t*_F_ are the saturation magnetization and the FM thickness, respectively. Below is the *J*_thr_ to align the magnetization in the in‐plane direction without FLT:^[^ ^5^ ^]^

(2) $$J_{\text{thr},\text{ DLT}}=\frac{1}{2}\frac{H_{k}}{h_{D}}$$

**Figure 1:**
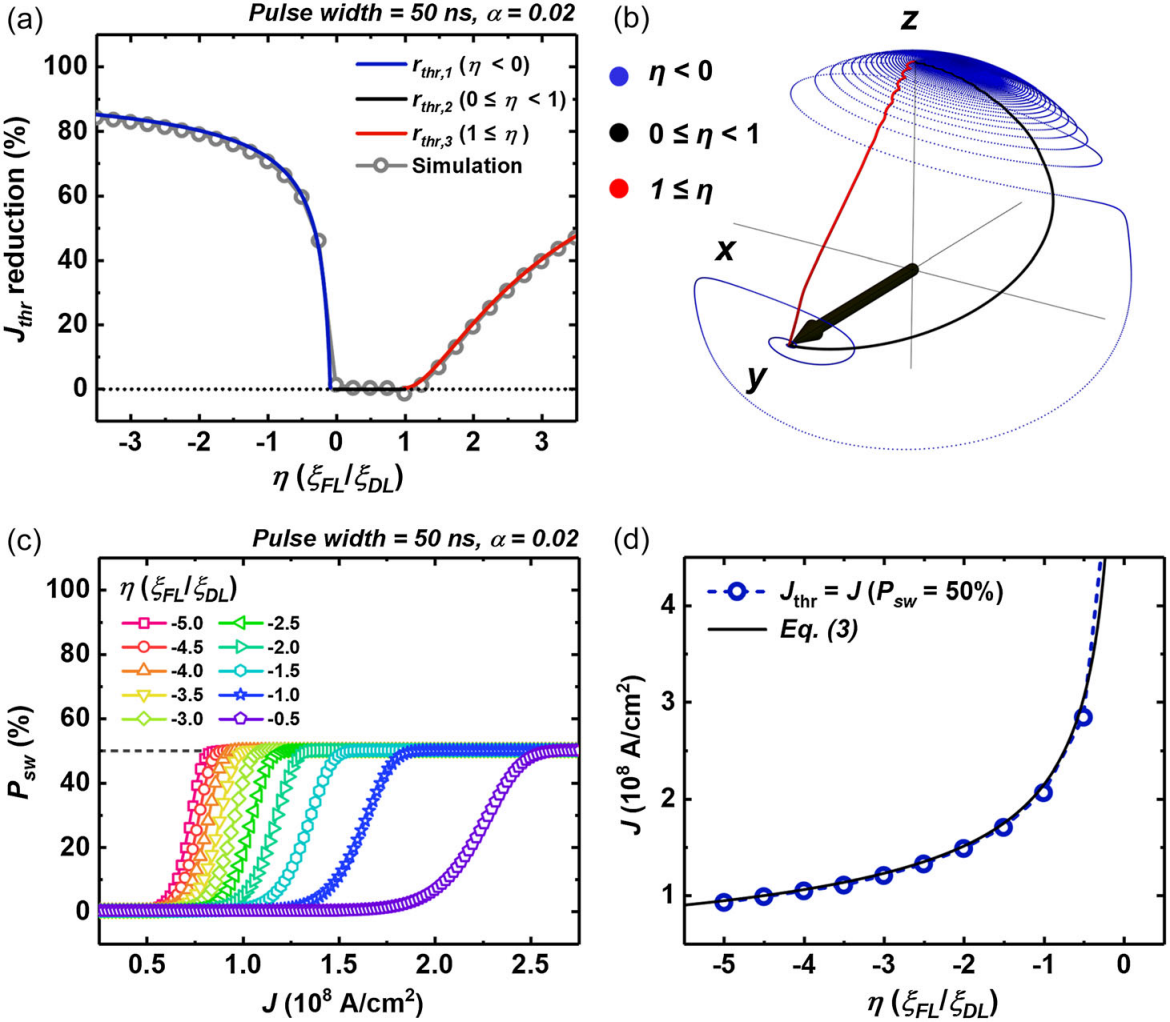
a) FLT‐induced threshold current density (*J*_thr_) reduction compared to the *J*_thr,DLT_ when the only factor exerting the switching is DLT. Each solid line shows the *J*_thr_ reduction from the different equation according to *η*: 1) *η* < 0 (blue solid line), 2) 0 ≤ *η* < 1 (black), and 3) 1 ≤ *η* (red). The gray open circles describe the *J*_thr_ reduction simulated by the LLG equation. The pulse width of each simulation is 50 ns with a rise time of 2 ns. b) The magnetization aligning trajectories induced by the FLT and DLT. Depending on the *η*, magnetization shows different behaviors: 1) *η* < 0 (blue solid line), 2) 0 ≤ *η* < 1 (black), and 3) 1 ≤ *η* (red). c) Fokker–Planck calculation of switching probability (*P*_sw_) with −5 ≤ *η* ≤ −0.5. The pulse width of the charge current flowing into NM is 50 ns with a 2 ns rise time. d) *η*‐dependent *J*_thr_, where the switching probability saturates to 50% for each *η*. The black solid line shows the threshold current density defined in Equation (3).

We note that SOT‐based RNG does not require an external field along the *x*‐direction, as it necessitates random switching along the +*z* or −*z* directions to facilitate random bit generation. In the presence of FLT, we also introduce three threshold current densities (*J*_thr*,i*_) to align the magnetization in the in‐plane direction shown below:^[^ ^5^, ^30^, ^33^ ^]^

(3) $$J_{\text{thr},1}=\sqrt{\frac{2\alpha }{\eta \left(\alpha \eta -2\right)}}\frac{H_{k}}{h_{D}},\;(\eta <0)$$

(4) $$J_{\text{thr},2}=\frac{1}{2}\frac{H_{k}}{h_{D}},\;\left(0\leq \eta <1\right)$$

(5) $$J_{\text{thr},3}=\frac{1}{1+\eta^{2}}\frac{H_{k}}{h_{F}},\;\left(1\leq \;\eta \right)$$

where *i* (= 1, 2, 3) represents the region according to the FLT‐to‐DLT ratio, *η* (1 for *η* < 0, 2 for 0 ≤ *η* < 1, and 3 for 1 ≤ *η*). We can easily calculate FLT‐induced *J*_thr_ reduction by defining $r_{\text{thr},i}=\left(J_{\text{thr},\text{DLT}}-J_{\text{thr},i}\right)/J_{\text{thr},\text{DLT}}$:

(6) $$r_{\text{thr},1}=1-2\sqrt{\frac{2\alpha }{\eta \left(\alpha \eta -2\right)}},\;(\eta <0)$$

(7) $$r_{\text{thr},2}=0,\;(0\leq \eta <1)$$

(8) $$r_{\text{thr},3}=\frac{{\left(1-\eta \right)}^{2}}{1+\eta^{2}},\;\left(1\leq \;\eta \right)$$

where the $r_{\text{thr},i}$ is a function of *α* and *η* only. **Figure** **1**a depicts a discernible decrease in the *J*_thr_ attributable to the influence of FLT as *η* varies while maintaining *α* = 0.02. Compared to *J*_thr, DLT_, the *J*_thr_ experiences a notable reduction as the dominance of FLT is amplified within the operational framework. When 1 ≤ *η*, the presence of FLT results in a reduction of the *J*_thr_. This reduction is facilitated by the assistance of the FLT over the DLT, which has a competitive interaction with the torque arising from the effective influence of PMA. Conversely, when η<0 is established, FLT introduces an anti‐damping torque component to the magnetization. This anti‐damping torque effect is responsible for the precessional trajectory of $\hat{m}$ observed in the blue solid line depicted in Figure 1b. This trajectory deviates from both the black solid line (corresponding to 0 ≤ *η* < 1) and the red solid line (1 ≤ *η*) in terms of the precessional magnetization behavior. Due to antidamping dynamics, the reduction in current density occurs with greater ease in instances where *η* < 0, as compared to the case of 1 ≤ *η*. Specifically, a 50% decrease from *J*_thr,DLT_ necessitates a magnitude of |*η*| equal to 0.32 in cases of *η* < 0, |*η*| amounting to 3.7 is requisite when considering 1 ≤ *η*. Given the anticipated more pronounced effect of FLT‐induced reduction under the conditions of *η* < 0, we focus on SOT‐based TRNG within the region. As described in Equation (6), *J*_thr_ can be reduced by lowering the damping parameter *α* or increasing the magnitude of *η*. We further focused on manipulating *η* rather than *α* because the lowering *α* is limited by enhanced damping from spin pumping in thin film FM/HM bilayer structure.^[^ ^34^, ^35^, ^36^, ^37^ ^]^

We performed Fokker–Planck calculation with macrospin approximation^[^ ^38^, ^39^, ^40^, ^41^, ^42^, ^43^ ^]^ to support the TRNG availability of SOT devices, including FLT. The Fokker–Planck equation numerically predicts the switching probability (*P*_sw_) of SOT switching, and we demonstrated the calculation details in Section S1, Supporting Information. Displayed in Figure 1c are the *P*_sw_ distributions corresponding to distinct values of *η*, ranging from −5 to −0.5, with increments of 0.5. The depicted data unequivocally reveal that the switching probability attains saturation at 50% across all examined *η* values. This observation demonstrates the viability of utilizing a SOT device featuring FLT for TRNG. Figure 1d made a comparison between the calculation result from Equation (3)^[^ ^30^ ^]^ (represented by the black solid line) and *J*_thr_ values (open symbols) obtained from the points where *P*_sw_ reaches saturation in Figure 1c. This result demonstrates a notable alignment between the outcomes yielded by the Fokker–Planck calculation and those obtained from the LLG equation.

### 2.2 Modulation of the FLT‐to‐DLT Ratio in NM–FM Heterojunctions

**Figure 2:**
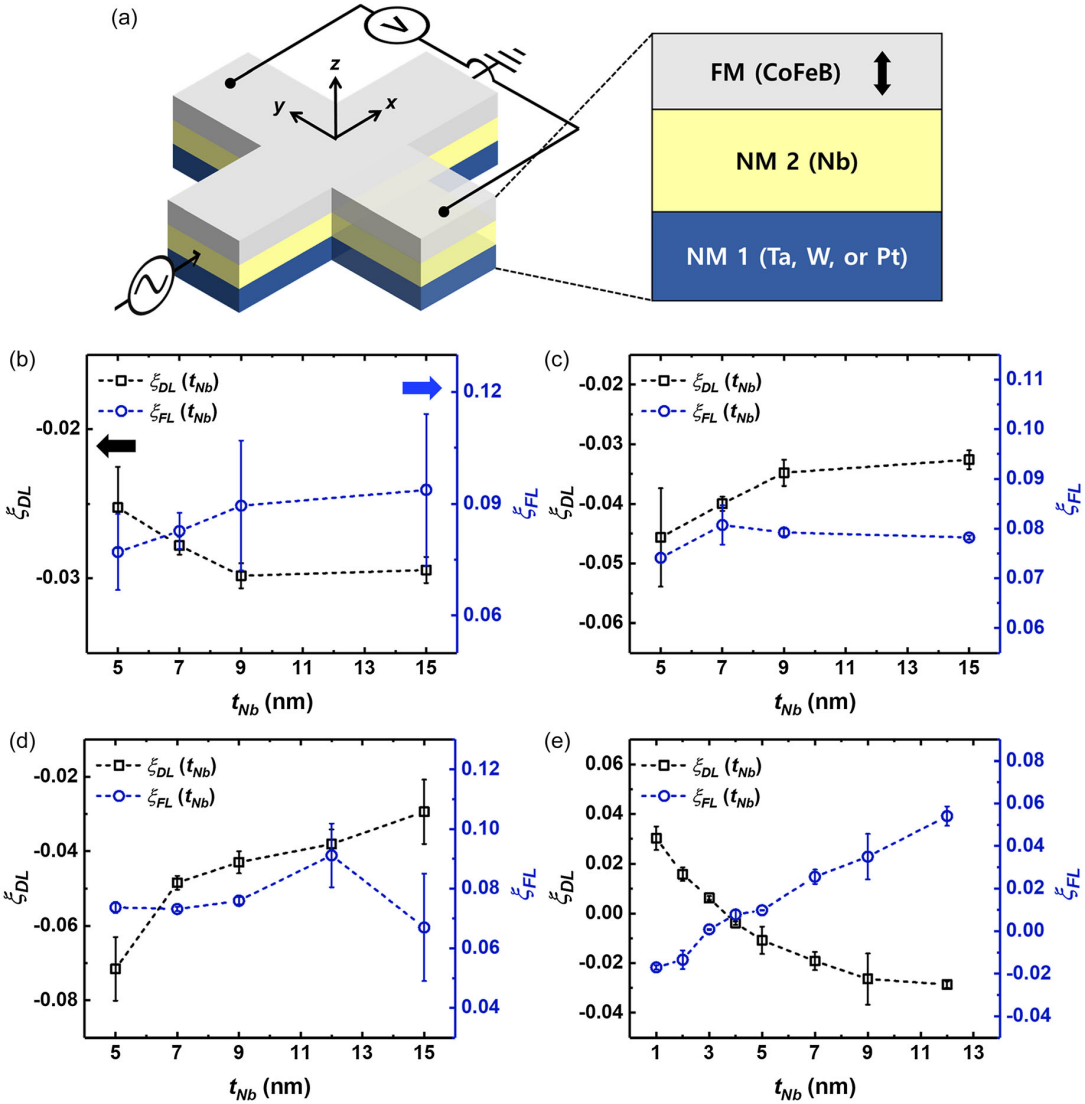
a) Schematic of trilayered Hall bar structure and measurement geometry. The line width for both current and voltage is 5 μm. b–e) DLT (*ξ*_DL_) and FLT efficiency (*ξ*_FL_) of the Nb series (b), Ta/Nb series (c), W/Nb series (d), and Pt/Nb series (e). The black (blue) points depict *ξ*_DL_ (*ξ*_FL_) of each series.

To verify theoretical predictions experimentally, we modified the thickness of the Nb layer by introducing NM 1/NM 2/CoFeB (NM 1 is W, Ta, or Pt, and NM 2 is Nb) heterojunctions. The Nb/CoFeB heterojunctions and those with Ta and W NM 1 layers consisted of different *t*_Nb_ as 5–15 nm. However, the series with Pt NM 1 layers employed 1–15 nm of Nb layers (see Experimental Section). All the series exhibited PMA confirmed from the *M*–*H* curves measured using vibrating sample magnetometry (Section S2, Supporting Information). We fabricated all PMA films into the 5 μm width Hall bar structure, as shown in **Figure** **2**a. We characterized the magnitude of effective SOT fields using the first and second Hall voltages (Section S3, Supporting Information). We defined the sign of each torque following Equation (S12) (Section S3, Supporting Information). *ξ*_FL_ > 0 demonstrates that Δ*H*_T_ is applying in the same direction as the Oersted field. Figure 2b–e depicts the efficiency of DLT and FLT for each Nb‐based series. The *ξ*_DL(FL)_ analyses conducted on heterojunctions comprising Nb/CoFeB demonstrate a gradual upward tendency, corresponding to the increase of *t*_Nb_, followed by subsequent attainment of saturation. This outcome is consistent with the forecasts made by the spin diffusion model. From the spin diffusion model perspective, a way to change *η* is to adjust the thickness of the spin current source layer (*t*_NM_) because the magnitude of the two torques increases as the *t*_NM_ increases when it does not reach the spin diffusion length. However, torques in that thickness region are insufficient to affect the SOT‐induced switching process. Only when *t*_NM_ exceeds a certain thickness (≈ a few nanometers), the spin currents can transfer sufficient torque for switching, but *η* already represents a fixed value, as shown in Figure 2b.^[^ ^6^, ^44^, ^45^ ^]^ Therefore, PMA devices operating via current‐induced SOT switching always have the same *η* depending on the material combination in the NM/FM heterostructure.

The behavior exhibited by the Ta, W/Nb series diverges significantly. The *ξ*_DL_ experiences an exponential reduction with increasing *t*_Nb_, whereas *ξ*_FL_ remains unaffected by variations in *t*_Nb_. In the Pt/Nb series, *ξ*_DL_ (*ξ*_FL_) exhibits a positive (negative) sign known that a Pt/FM heterojunction possesses when *t*_Nb_ is lower than 4 nm. Over the 4 nm, *ξ*_DL_ (*ξ*_FL_) within Pt/Nb series experience a sign reversal, exhibiting a progressive increase followed by eventual saturation comparable to that observed in the Nb/CoFeB. We also confirmed competing spin currents by conducting the deterministic SOT switching measurement under a 200 Oe external field (Section S4, Supporting Information). The role of the NM 2 layer involves serving as the source and channel for spin current within these heterojunctions. Consequently, an anticipated decline in the DLT is linked to the amplification of *t*_NM2_, due to the decrease in the spin current originating in the NM 1 layer that reaches the magnetic layer. Nonetheless, while the majority of the DLT originates from the spin Hall effect taking place in bulk heavy metals, it is known that the interfacial spin–orbit coupling influences the FLT.^[^ ^46^, ^47^, ^48^ ^]^

**Figure 3:**
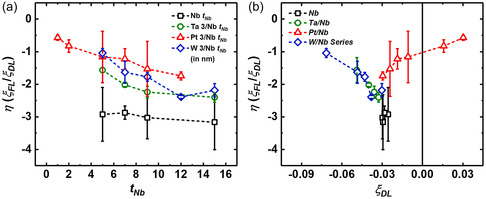
a,b) *η* = *ξ*_FL_/*ξ*_DL_ of the NM 1/NM 2/FM heterojunctions as a function of NM2 thickness (a) and DLT efficiency (*ξ*_DL_) (b). The values of *ξ*_DL_ and *ξ*_FL_ were derived from the data presented in Figure 2b–e. The ratio, *η*, was calculated by dividing their respective values.

Our previous study demonstrated distinct rates of torque alteration concerning *t*_NM2_ within the heterojunctions.^[^ ^10^ ^]^ Accordingly, the *η* of the devices exhibited a range from 0.5 to 3 in magnitude, as shown in **Figure** **3**a,b. Notably, the Nb/CoFeB heterojunctions displayed an FLT to DLT ratio 3 times as significant, constituting the most substantial ratio within our structures, irrespective of *t*_Nb_. Trilayered heterojunctions demonstrated a diminishing *η* as *t*_Nb_ decreases. This decline can be attributed to the heightened strength of spin–orbit coupling in the structures comprising NM 1 relative to Nb, thereby contributing to an enhanced *ξ*_DL_ within devices featuring a thin Nb layer. However, a variation existed in the extent of *ξ*_FL_ alteration compared to *ξ*_DL_, leading to a progressive reduction in *η*. The Ta/Nb and W/Nb series reach their saturation point when the Nb layer surpasses 12 nm. In the Pt/Nb series, the *η* value is further reduced owing to the favorable positive sign of spin–orbit coupling associated with Pt, in contrast to the negative sign characteristic of Nb, Ta, and W. The competing spin currents generated by the Pt and Nb tend to counterbalance the cumulative influence of each other within the range of 3 < *t*_Nb_ < 4 nm, as shown in Figure 2e. Notably, the favorable positive sign of DLT becomes prominent when *t*_Nb_ falls below 3 nm in the Pt/Nb series. Nevertheless, the product of signs between FLT and DLT consistently displays negativity across all devices. These outcomes correspond with experimental observations made in traditional NM/FM heterostructures and investigations aimed at manipulating DLT and FLT.^[^ ^10^, ^46^, ^49^, ^50^ ^]^

### 2.3 Correlation of the Threshold Current Density with the FLT‐to‐DLT Ratio

**Figure 4:**
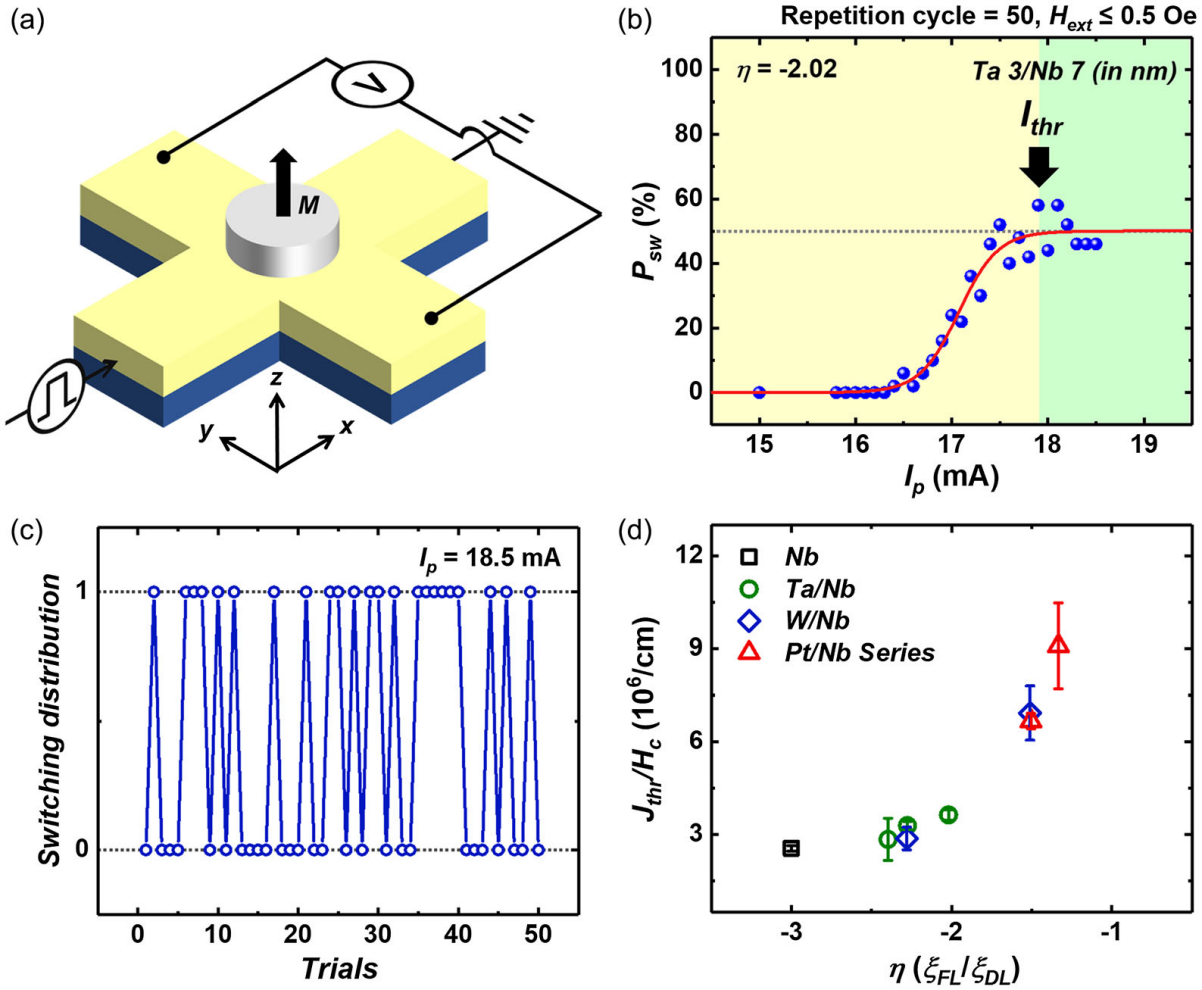
a) Schematic of current‐induced SOT switching measurement system. While we inject the current pulse, the magnetization lies in the in‐plane direction, and when stopping the current supply, it aligns randomly in the +*z* or −*z* direction. The aligning direction was characterized by reading the transverse Hall voltage. b) Switching probability (*P*_sw_) distribution of Ta 3/Nb 7/CoFeB 1/MgO 1/Ta 2 (in nm); *η* = −2.02. The blue dots show the probability of switching occurring when performing 50 write operations for each current level. The red line is a fitting curve using the Boltzmann sigmoidal function. c) Switching distribution when we applied 18.5 mA for 50 trials. d) Threshold current density (*J*_thr_) normalized by coercive field (*H*_c_) as a function of *η*. *J*_thr_/*H*_c_ is reduced exponentially as the FLT dominates the system.

We measured the current‐induced SOT switching using Nb/CoFeB and three types of NM (Ta, W, and Pt)/Nb/CoFeB heterojunctions. The 5 μm‐in‐diameter dot‐shaped FM layer was fabricated for the measurement, as shown in **Figure** **4**a. We obtained effective magnetization reversal boosted by SOT, and its switching polarity reversed depending on the external field direction, well known as evidence of SOT switching. After assuring the switching operation, we induced a current pulse (*I*_p_) without an external field to observe the *P*_sw_ (Section S5, Supporting Information). Figure 4b shows the typical *P*_sw_ curve of the SOT device (Ta 3/Nb 7/CoFeB 1/MgO 1/Ta 2 in nm) as a function of *I*_p_. The blue points represent the number of times the magnetization reversal occurs when injecting the corresponding *I*_p_ 50 times. When applying insufficient current, the spins do not entirely lie down to the in‐plane direction and return to their first position (*P*_sw_ = 0). *P*_sw_ increases as the *I*_p_ increases and saturates to 50%. We use the Boltzmann sigmoidal function to extract the threshold current (*I*_thr_) by setting the boundary value 0.5 as a maximum and 0 as a minimum as follows:

(9) $$P_{\text{sw}}=0.5\left(1-\frac{1}{1+e^{\left(x-x_{0}/dx\right)}}\right)$$

We experimentally defined the *I*_thr_ as the *x* value, resulting in *P*_sw_ = 0.49. The measured *I*_thr_ of the device is 17.8 mA, beyond which the *P*_sw_ saturates to 50%. The probabilities of all SOT devices approach 50% as the *I*_p_ magnitude increases (Figure S6, Supporting Information). Figure 4c illustrates the distribution of switching behavior when a pulse of 18.5 mA is applied to the device 50 times. Across the 50 pulse injections, switching occurred 23 times, indicating a probability of 46%.

In developing practical spintronic devices, the switching current (*I*_sw_) is essential for determining energy efficiency and endurance ($\propto I_{\text{sw}}^{2}$).^[^ ^33^ ^]^ Therefore, we graphed the *I*_thr_ and *J*_thr_ attained in the switching probability experiment against *η* (Section S6, Supporting Information). The extracted *I*_thr_ did not show a tendency according to *η* of each device, but *J*_thr_ showed a weak tendency to decrease as *η* increased, as theoretically expected. However, DLT and FLT are not the only factors affecting the switching process. Accordingly, we can simplify the representation of the *J*_sw_ by employing the following equations, considering a domain wall propagation model:^[^ ^28^, ^33^ ^]^

(10) $$J_{\text{sw}}\;=\frac{4e\mu_{0}M_{s}t_{F}H_{c}}{\pi \hbar \theta_{\text{SH}}}$$

where *M*_s_ is the saturated magnetization (kA m^−1^), *t*_F_ is the thickness of the free layer, *θ*_SH_ is the spin Hall angle, and *H*_c_ is the coercive field (A m^−1^), respectively. *J*_sw_ is inversely proportional to the *θ*_SH_ but proportional to *H*_c_. The coercivity value of the NM/FM heterojunctions, which exhibit PMA, is affected by the combination of materials employed and the corresponding interface conditions. Given the diversity in material combinations within our samples across distinct series, variations in *H*_c_ are observed among different devices within each series (Section S7, Supporting Information). We calculated *J*_thr_/*H*_c_ to examine the only impact of *η* on SOT switching efficiency, as shown in Figure 4d. Notably, the efficiency of SOT operation is enhanced when *ξ*_FL_ surpasses *ξ*_DL_. Given the distinct torque actuation mechanisms, this outcome aligns well with theoretical expectations. Among our devices, the Nb/CoFeB heterostructures with the highest *η* demonstrate the most effective SOT switching process despite not having the largest *ξ*_DL_. Conversely, the Pt/Nb series, characterized by a low *η*, necessitates a higher current density despite having a similar *ξ*_DL_ compared to other series. These findings indicate that DLT efficiency is not the sole determinant of SOT device operation, underscoring the importance of appropriately considering FLT.

### 2.4 Reliability of High FLT‐Based TRNGs

**Figure 5:**
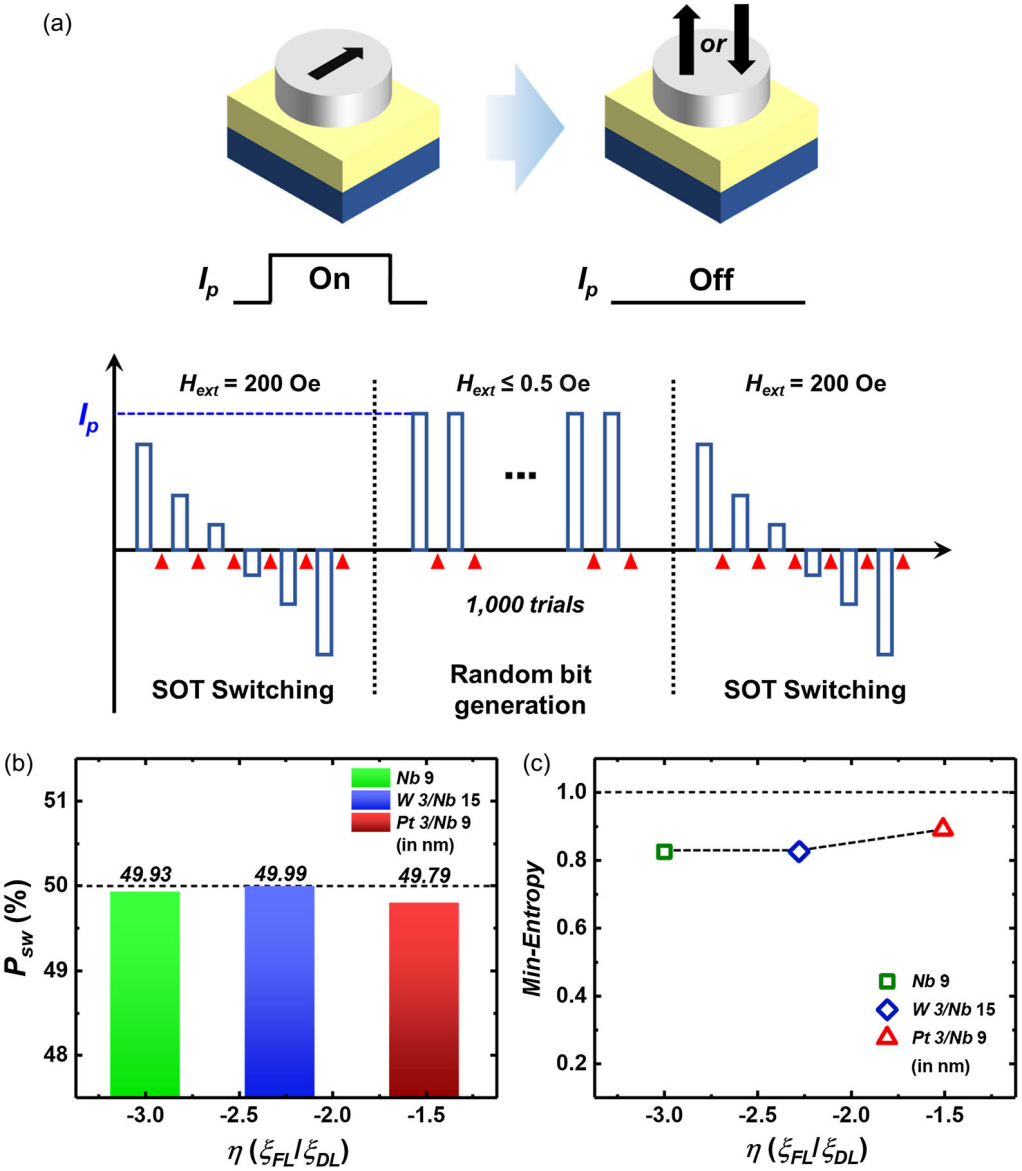
a) A sequence of random number generation using a trilayer device. First, we assured deterministic‐SOT switching with an external magnetic field of 200 Oe. After that, we turned off the external magnetic field, injected a sufficient pulse (*I*_p_) enough to align magnetization in‐plane with a length of 10 μs (*I*_p_ is different for each device) 1000 times, and measured the Hall voltage after each injection. After 1000 write operations, we performed the postdeterministic‐switching measurement to check the state of the device to confirm the validity of the random string. The open blue rectangle represents the applied pulse, while the red triangles signify measurements of the Hall voltage. b) Switching probability (*P*_sw_) for the SOT devices exhibiting distinct values of parameter *η*. c) Min‐entropy obtained from devices characterized by different *η*, assessed through the NIST SP 800‐90B test.

As shown above, we experimentally validated that in NM/FM heterojunction structures exhibiting PMA, operational currents can be reduced when FLT predominates over DLT. Here, we discuss whether SOT devices dominated by FLT can sustain the ability to generate random numbers. We use three SOT devices with different *η* values—device #1: Nb 9/CoFeB 1 nm (*η* = −3.03), device #2: W 3/Nb 15/CoFeB 1 nm (*η* = −2.18), and device #3: Pt 3/Nb 9/CoFeB 1.1 nm (*η* = −1.53). **Figure** **5**a depicts a sequence of random number‐generating processes. Initially, we induced deterministic SOT switching by applying an external field along the current injection direction. Subsequently, an external magnetic field is turned off to align the magnetization in the in‐plane direction, followed by injecting a 10 μs‐long *I*_p_ (write step). Immediately following this, the measurement of the Hall resistance (*R*_H_) allows for the determination of whether the magnetization is oriented toward the “up” or “down” direction (read step). Each instance of the write–read sequence yields one bit, and this sequence was repeated 1000 times. We conducted deterministic SOT switching following 1000 cycles of write–read operations to validate the reliability of random bits generated from functional SOT devices. Damage or artifacts introduced during measurement were adjusted based on the *R*_H_ obtained in postswitching steps, and subsequent counting 0 and 1 s were performed (Section S8, Supporting Information). Note that the experiment system obeys the multidomain regime, resulting in nonuniform *R*_H_ distribution. Nevertheless, as measured values fall within the observed switching variation, the net magnetization can effectively differentiate between 0 and 1.

The number of bits generated is as follows for each device: device #1 (*η* = −3.03) with 308 683 bits, device #2 (*η* = −2.18) with 188 406 bits, and device #3 (*η* = −1.53) with 392 156 bits. Figure 5b depicts *η*‐based switching probability. Proposed SOT‐TRNGs demonstrate nearly 50% switching probability, irrespective of *η*. We conducted the NIST 800‐90B test to confirm the randomness of the random number sequence obtained from the above three devices. The entropy estimation test suite is specifically designed to assess the entropy source of TRNGs, focusing on verifying the unpredictability. As the proposed SOT‐TRNG is based on a nondeterministic process, it is suitable to employ NIST SP 800‐90B to examine the statistical characteristics. The entropy evaluation process involves determining the track (IID or non‐IID) and estimating the entropy of the raw random sequence. For IID sequences (independent and identically distributed), entropy is evaluated using the most common value estimation. However, for non‐IID sequences (like ours), entropy is assessed using the minimum value (min‐entropy) from ten entropy estimates. Generating an m‐bit random number sequence requires bit generation at the *m*/*n* scale, where *n* (0 ≤ *n* ≤ 1) represents the min‐entropy value. For an ideal TRNG, the desired min‐entropy value is 1, indicating optimal randomness.^[^ ^51^, ^52^ ^]^ **Table** **1** shows the result of the test. The values written in bold represent the lowest entropy values among the ten estimators. We verified no trend in min‐entropy values for *η,* as shown in Figure 5c. This result shows that the bit data generated in the NM 1/NM 2/FM heterojunctions, where FLT is relatively dominant (*η* < −1), has randomness regardless of the value of *η* and demonstrates that we can consider the SOT device as a TRNG application.

**Table 1:** NIST SP 800‐90B test with random bits generated by SOT devices

Estimator	Device #1 (Nb 9) (*η* = −3.03)	Device #2 (W 3/Nb 15) (*η* = −2.18)	Device #3 (Pt 3/Nb 9) (*η* = −1.53)
MCW	0.991272	0.989727	0.987808
Collision	1.000000	0.857758	1.000000
Markov	0.980641	0.995651	0.966631
Compression	**0.825407** a)	**0.825778** a)	1.000000
t‐Tuple	0.852528	0.871707	**0.891471** a)
LRS	0.961656	0.935949	0.943386
MultiMCW	1.000000	1.000000	1.000000
Lag	0.916287	0.978432	0.920157
MultiMMC	0.855569	0.924052	0.932909
LZ78Y	0.974511	0.991302	0.961145

a) The lowest entropy value (Min‐entropy) is highlighted in bold, representing the representative entropy for each device.

### 2.5 Demand for Systematic FLT‐Manipulating Studies in SOT Devices

Most simple NM/FM bilayer structures show *η* < 0 values.^[^ ^14^ ^]^ As of now, experimental validation has only been achievable within the parameter range where *η* is below 0. To comprehensively investigate FLT‐modulated SOT‐RNG, demonstrating the SOT device in the *η* > 0 regions is essential. The previous study shows that such devices can effectively reduce critical switching current.^[^ ^32^ ^]^ Section S9, Supporting Information, reveals that the *J*_thr_ of *η* > 0 SOT devices remain unaffected by pulse width and damping parameter, unlike *η* < 0 devices that exhibit increased *J*_thr_ for higher damping parameters or shorter pulse widths. Even in that region (*η* > 0), the magnetization undergoes motion along an uncomplicated and abbreviated trajectory, as shown in Figure 1b. Thus, it is valuable to demonstrate *η* > 0 SOT devices due to their advantages in fast operation and compatibility with high‐damping metals. NM/FM/NM trilayer or novel materials can be promising candidates for *η* > 0 SOT‐RNG.

## 3 Conclusion

This study controlled the FLT‐to‐DLT ratio by modifying the thickness and materials of the NM layer in the NM 1 (Ta, W, or Pt)/NM 2 (Nb)/FM (CoFeB) heterojunctions to investigate the FLT impact on SOT switching properties. We characterized switching probability by varying input currents. We demonstrated that the *η* ranges from −0.5 to −3, and the *J*_thr_ exponentially increased as the magnitude of *η* decreased, consistent with theoretical calculations. We confirmed that the energy required for switching decreases as the FLT influence on the system relatively increases. Furthermore, we generated the random numbers using the SOT device belonging to the *η* ≤ −1 region to confirm the feasibility of RNG through the NIST SP 800‐90B entropy test. Remarkably, regardless of *η*, our trilayers consistently produced validated random bits. Our results suggest that controlling *η* benefits SOT‐based TRNG with low energy consumption.

## 4 Experimental Section

4.1

4.1.1

##### Sample Preparation

We deposited all samples using DC and RF magnetron sputtering for metals and oxides, respectively, onto 300 nm thick thermally oxidized 1.25 × 1.25 cm^2^ Si wafers under a base pressure below 5 × 10^−9^ Torr. The reference film stacks consist of Si/SiO_2_/NM 1 (*t*_NM1_)/NM 2 (*t*_NM2_)/Co_40_Fe_40_B_20_ (1 or 1.1)/MgO (1)/Ta (2) (in nm), where NM 1 = Ta, W and NM 2 = Nb. The thickness of the Nb layer varied from 5 to 15 nm for the NM 1/NM 2 series, where the spin–orbit coupling signs of the two layers were equal. Nb ranged from 0 to 15 nm, and we fixed CoFeB to 1.1 nm for the series where NM 1 (= Pt) and NM 2 have opposite signs. All samples were postannealed at 300 °C for 1 h at a magnetic field of 6 kOe applied perpendicularly to the film plane under a base pressure of 10^−6^ Torr.

##### Device Manufacturing

We patterned Hall bar devices, 5 μm in width and 35 μm in length, using photolithography (MDA‐400M‐06, MIDAS) and Ar ion milling. Then, we deposited the electrode consisting of Ti (10)/Au (100) (in nm) by sputtering and formed using the lift‐off. We further fabricated dot‐shaped SOT devices using the same photolithography and ion milling system. The milling stopped at the NM/FM interface due to the design of the dot‐shaped structure consisting of a CoFeB/MgO/Ta layer with a 5 μm diameter.

##### Electrical Measurements

A 4‐point probe station (MSTECH M7VC) measured the electrical resistivity and current‐induced SOT switching. We applied current with a 10 μs pulse width and an external magnetic field parallel to the current direction during measurement. For the investigating threshold current density, we induced a current without an external field to observe the switching probability and repeated the following four steps 50 times to obtain probability properties—step 1: applying the external field in +*x* (−*x*) direction; step 2: inducing current to the +*x* (−*x*) direction to first set the magnetization in the +*z* (−*z*) direction; step 3: turning off the external field; step 4: inducing current to settle magnetization along the *y*‐axis. When we turned off the power supply of the electromagnet, the residual magnetic field applied to the device was 0.5 Oe or less. Switching was not secured when we conducted current‐induced SOT switching under the residual magnetic field. Thus, we considered that the field had a negligible effect on the system.

## Conflict of Interest

The authors declare no conflict of interest.

## Author Contributions

M.H.L., S.‐J.K., and S.I.Y. contributed equally to this work. M.H.L., K.‐J.L., and Y.K.K. conceived the study. M.H.L. and S.I.Y. designed experiments and analyzed the data. S.‐J.K. conducted the theoretical calculations and interpretation. M.H.L., J.K.L., and H.S.K. fabricated films. M.H.L. and S.I.Y. conducted SOT switching measurements and generated random numbers bit. G.K. and S.H. performed the NIST test for randomness verification. M.H.L., S.‐J.K., and S.I.Y. wrote the manuscript after a discussion with all the authors. K.‐J.L. and Y.K.K. supervised the entire project.

## Supporting information

Supplementary Material

## Data Availability

The data that support the findings of this study are available from the corresponding author upon reasonable request.
