# Supplementary material for: Reduction of Operating Current by Harnessing the Field‐ and Damping‐Like Torque Ratios in Nonmagnet–Ferromagnet Heterojunctions
Source: Small Sci. 2023 Dec 15;4(2):2300224. doi: 10.1002/smsc.202300224 (PMC11935181; doi:10.1002/smsc.202300224)
Supplement: Supplementary file 1 — Supplementary Material [file SMSC-4-2300224-s001.pdf]

Supporting Information

Reduction of Operating Current by Harnessing the Field-Like and Damping-Like Torque Ratios in Nonmagnet-Ferromagnet Heterojunctions

Min Hyeok Lee, Seok-Jong Kim, Seok In Yoon, Jeong Kyu Lee, Han Seok Ko, Gyusang Kim, Seokhie Hong, Kyung-Jin Lee, and Young Keun Kim**

M. H. Lee, S. I. Yoon, J. K. Lee, H. S. Ko, Prof. Y. K. Kim
Department of Materials Science and Engineering, Korea University, Seoul, 02841, Republic of Korea
E-mail: ykim97@korea.ac.kr (Prof. Y. K. Kim)

S.-J. Kim, Prof. K.-J. Lee
Department of Physics, Korea Advanced Institute of Science and Technology (KAIST), Daejeon 34141, Republic of Korea
E-mail: kjlee@kaist.ac.kr (Prof. K.-J. Lee)

S.-J. Kim
Department of Materials Science and Engineering, Korea Advanced Institute of Science and Technology (KAIST), Daejeon 34141, Republic of Korea

G. Kim, Prof. S. Hong
Institute of Cyber Security & Privacy (ICSP), School of Cybersecurity, Korea University, Seoul 02841, Republic of Korea

Section S1. Fokker-Planck calculation of switching probability

In this note, we numerically investigate the switching probability driven by field-like torque (FLT) with Fokker-Planck calculation. We introduce the Landau-Lifshitz-Gilbert (LLG) and the Fokker-Planck equations, respectively.

The LLG equation describes magnetization dynamics,

$$\dot{\hat{\mathbf{m}}} = -\gamma\hat{\mathbf{m}} \times \mathbf{H}_{\text{eff}} + \alpha\hat{\mathbf{m}} \times \dot{\hat{\mathbf{m}}} + \gamma c_j \hat{\mathbf{m}} \times (\hat{\mathbf{m}} \times \hat{\boldsymbol{y}}) + +\gamma\eta c_j \hat{\mathbf{m}} \times \hat{\boldsymbol{y}}\,, \tag{S1}$$

where the $\hat{\mathbf{m}}$ is the unit vector along the magnetization, γ is the gyromagnetic ratio, $\mathbf{H}_{\text{eff}} = H_k \hat{\mathrm{m}}_z \hat{\mathbf{z}}$ is the effective field, including the perpendicular anisotropy field at the amount of H_k, α is the Gilbert damping constant, c_j is an effective field of DLT defined as $\hbar\theta_D J/2eM_s t_f$, $\hbar$ is reduced Planck constant, θ_D is spin Hall angle, J is the charge current density flowing through the heavy metal layers, e is the electron charge, M_s is the saturation magnetization, t_F is the thickness of the magnetic layer, and η is the ratio of the magnitude of FLT to DLT. We can write down Eq. (S1) in the spherical coordinate using $\dot{\hat{\mathbf{m}}} = \frac{\partial\theta}{\partial t}\hat{\boldsymbol{\theta}} + sin\theta\frac{\partial\phi}{\partial t}\hat{\boldsymbol{\phi}}$,

$$\tau_D\left(\frac{d\theta}{dt}\right) = \mathbf{h}_{\text{eff}} \cdot \left(\frac{1}{\alpha}\hat{\boldsymbol{\phi}} + \hat{\boldsymbol{\theta}}\right) - j\hat{\mathbf{y}} \cdot \left\{(1+\eta\,\alpha)\hat{\boldsymbol{\theta}} + (\eta - \alpha)\hat{\boldsymbol{\phi}}\right\} \quad , \tag{S2}$$

$$\tau_D\left(sin\theta\frac{d\phi}{dt}\right) = \mathbf{h}_{\text{eff}} \cdot \left(-\frac{1}{\alpha}\hat{\boldsymbol{\theta}} + \hat{\boldsymbol{\phi}}\right) - j\hat{\mathbf{y}} \cdot \left\{(1+\eta\,\alpha)\hat{\boldsymbol{\phi}} - (\eta - \alpha)\hat{\boldsymbol{\theta}}\right\}, \tag{S3}$$

where θ and ϕ are the polar and azimuthal angles of $\hat{\mathbf{m}}$, $\tau_D = (1+\alpha^2)/\alpha\gamma H_k$, $\mathbf{h}_{\text{eff}} = \mathbf{H}_{\text{eff}}/H_k = cos\,\theta\,\hat{\mathbf{z}}\,,\ and\ j = c_j/\alpha H_k$.

The Fokker-Planck equation can be driven for the probability distribution of the polar angle θ and azimuthal angle ϕ as a function of time t[S2-S6]. We define $\mathrm{W}(\theta, \phi, \mathrm{t})$ as the probability that the spherical coordinate of magnetization at time t is (θ , ϕ). $\mathrm{W}(\theta, \phi, \mathrm{t})$ follows the normalization condition that the sum of the probability distribution is constant as 1,

$$\iint \mathrm{W}(\theta, \phi, \mathrm{t})\, sin\theta d\theta d\phi = 1, \tag{S4}$$

$\mathrm{W}(\theta, \phi, \mathrm{t})$ also follows the Fokker-Planck equation written in the form of a continuity equation,

$$\frac{\partial \mathrm{W}}{\partial \mathrm{t}} = -\nabla \cdot \mathbf{J}(\theta, \phi), \quad (S5)$$

$$\nabla \cdot \mathbf{J}(\theta, \phi) = \frac{1}{sin\theta}\left\{\frac{\partial(\sin\theta\, J_\theta)}{\partial\theta} + \frac{\partial J_\phi}{\partial\phi}\right\}, \quad (S6)$$

$$J_\theta = \frac{\partial\theta}{\partial \mathrm{t}}W - D\frac{\partial \mathrm{W}}{\partial\theta},\ \ J_\phi = \sin\theta\frac{\partial\phi}{\partial \mathrm{t}}W - D\frac{1}{sin\theta}\frac{\partial \mathrm{W}}{\partial\phi}, \quad (S7)$$

where D is the diffusion coefficient derived as $D = \alpha\gamma k_B T/(1+\alpha^2)M_s V$,[S6] where V is the volume of the magnetization unit cell, and T is the temperature. We combine Eqs. (S2-S3) and Eq. (S5) above, as shown below,

$$\tau_D \frac{\partial W(\theta,\phi)}{\partial t} = -\frac{1}{sin\theta}\frac{\partial}{\partial\theta}\left[sin\theta\left\{\{-\sin\theta\cos\theta - j\big((1+\eta\,\alpha)\sin\phi\cos\theta + (\eta-\ \alpha)\cos\phi\big)\}W - \frac{1}{2\Delta}\frac{\partial W}{\partial\theta}\right\}\right]$$

$$-\frac{1}{sin\theta}\frac{\partial}{\partial\phi}\left[\left\{\frac{1}{\alpha}\cos\theta\sin\theta - j\big((1+\eta\,\alpha)\cos\phi - (\eta-\ \alpha)\sin\phi\cos\theta\big)\right\}W - \frac{1}{2\Delta}\frac{1}{sin\theta}\frac{\partial W}{\partial\phi}\right], \quad (S8)$$

where Δ is the thermal stability factor defined as $\Delta = H_k M_s V/2k_B T$.[S5] The simulation result of Eq. (S8) is shown in Figure 1c in the manuscript. We define magnetization switching probability P_{sw} as the sum of $\mathrm{W}(\theta, \phi, t)$ at $\theta \geq \pi/2$,

$$P_{sw} = \iint_{\pi/2}^{\pi} \mathrm{W}(\theta, \phi, \mathrm{t})\, sin\theta d\theta d\phi. \quad (S9)$$

P_{sw} is calculated at various charge current densities J with a pulse width of 50 ns and rise-fall time of 2 ns for each η. Other parameters for simulations are: $H_k = 2000$ A/m, $\alpha = 0.02, \theta_D = -0.04, 0 \leq J \leq 3\times10^8 \mathrm{A/cm^2}$, $M_s = 1000$ kA/m, $t_f = 1$ nm, $-5 \leq \eta \leq 0,\ V = 30\times30\times1\ \mathrm{nm^3}, T = 300$ K.

Section S2. Perpendicular magnetic anisotropy of Nb/CoFeB/MgO/Ta and NM/Nb/CoFeB/MgO/Ta

The magnetic properties of Nb (t_{Nb})/$Co_{40}Fe_{40}B_{20}$ (1)/MgO (1)/Ta (in nm) films in the in-plane and perpendicular field direction using a vibrating-sample magnetometer (Microsense EV9), as shown in Figure S1. Normalized magnetization of the films was characterized by saturation magnetization (M_s). All samples show PMA regardless of t_{Nb}. The magnetic properties of NM 1 (3)/NM 2 (t_{Nb})/FM trilayer structure (NM 1 = Ta, Pt, or W, NM 2 = Nb, FM = CoFeB) were also measured, as shown in Figure S2. All trilayer samples exhibit PMA. In Ta/Nb and W/Nb series, t_{Nb} was 5, 7, 9, 12, and 15 nm. In the Pt/Nb series, t_{CoFeB} = 1.1 nm, t_{Nb} varied as 1, 2, 3, 4, 5, 7, 9, 12, and 15 nm. The insets show the whole range of out-of-plane hysteresis curves.

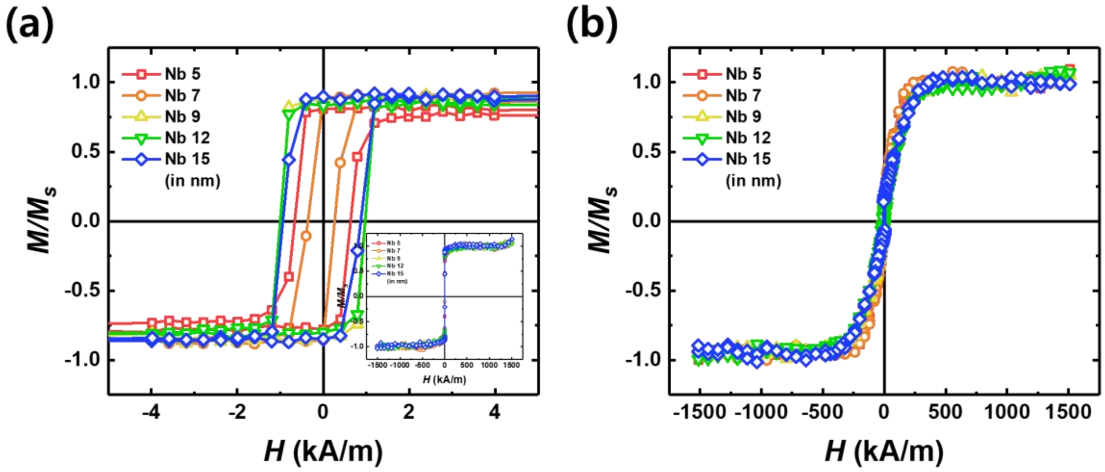

Figure S1. Normalized magnetization of Nb/CoFeB/MgO/Ta (a) out-of-plane and (b) in-plane normalized hysteresis loop of Nb (t_{Nb})/CoFeB 0.9/MgO 1/Ta 2 (in nm). Inset in (a) shows the full loops of out-of-plane.

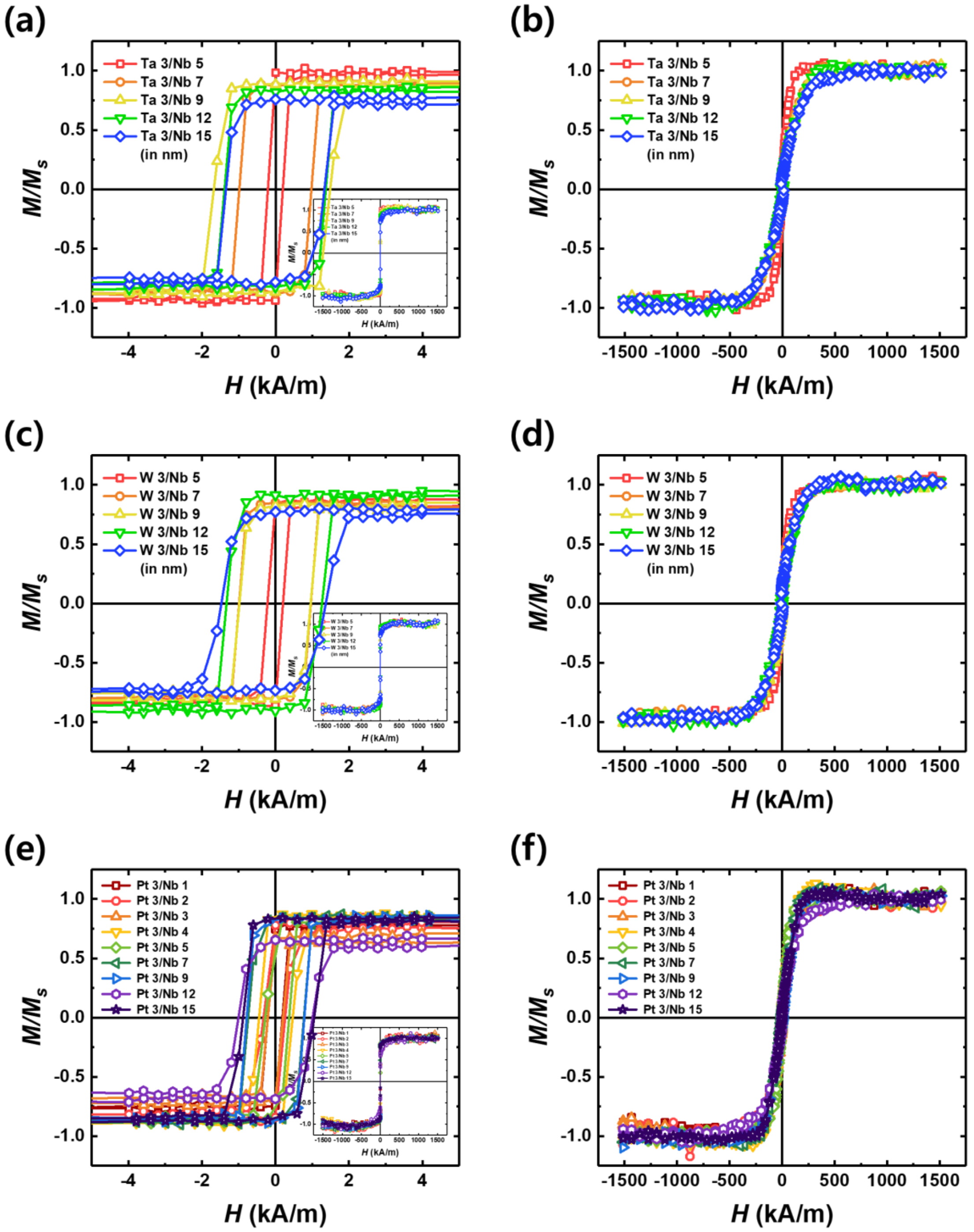

Figure S2. Normalized magnetization of NM/Nb/CoFeB/MgO/Ta (a), (c), (e) out-of-plane and (b), (d), (f) in-plane normalized hysteresis loop of trilayer structures. Insets of (a), (c), and (e) show the entire loop of out-of-plane.

Section S3. The harmonic signals of Nb-based heterostructures

To measure the SOT, we patterned Hall bar devices, 5 μm in width and 35 μm in length, using photolithography. Then, we deposited the electrode consisting of Ti (10)/Au (100) (in nm) by sputter and formed using the lift-off. The Hall bar devices were wire-bonded and placed on the stage with motors that could rotate the device along the polar (θ) and azimuthal (φ) angles.

We used 1-3 mA alternating current flowing into the *x*-direction and a fixed frequency of 13.7 Hz to the wired device. During the measurement, the external field sweeps up to 18 kOe. We measured the first harmonic ($V_{1\omega}$) and second harmonic Hall voltage ($V_{2\omega}$) with lock-in amplifiers (Stanford Research System SR830) while sweeping the external in-plane field. We swept the external in-plane field along ±*x* (±*y*) direction to observe the longitudinal (transverse) derivative ratio $B_{x(y)} = \left(\frac{\partial V_{2\omega}}{\partial H_{L(T)}}\right) \Big/ \left(\frac{\partial^2 V_{1\omega}}{\partial H_{L(T)}^2}\right)$. Subsequently, we extract the effective longitudinal field $\varDelta H_L$ (transverse field $\varDelta H_T$), which exerts DLT (FLT), using the following equation.[S1]

$$\varDelta H_{L(T)} = -2\left(B_{x(y)} \pm 2\xi B_{y(x)}\right)/(1 - 4\xi^2) \tag{S10}$$

The parameter ξ is the ratio of the PHE and AHE resistances. SOT efficiency ($\xi_{DL(FL)}$) can be obtained from the below equation.

$$\xi_{DL(FL)} = \frac{2e}{\hbar}\frac{M_s t_F \varDelta H_{L(T)}}{j_c} \tag{S11}$$

We can express the total energy equation for the NM/FM bilayer structure, including $\varDelta H_L$ and $\varDelta H_T$ as follows,

$$E_{tot} = -K_{eff}\,(\hat{\mathbf{m}} \cdot \hat{\mathbf{z}})^2 - M_s \hat{\mathbf{m}} \cdot (\boldsymbol{H_{ext}} - \varDelta H_L \hat{\mathbf{m}} \times \hat{\mathbf{y}} - \varDelta H_T \hat{\mathbf{y}}) \tag{S12}$$

K_{eff} is the effective PMA energy, M_s is saturation magnetization, and H_{ext} is an external field.

The first column in Figure S3 (S3a, S3d, S3g, and S3j) is the first harmonic signal of each sample series. The second (S3b, S3e, S3h, and S3k) and third columns (S3c, S3f, S3i, and S3l) show the second harmonic signals with x-direction external field and y field, respectively. Nb/CoFeB heterojunctions and Nb-based trilayers consisting of the same SOC signs between NM 1 and NM 2 (Ta, W/Nb/CoFeB) exhibited negative DLT (positive FLT) sign according to

our sign definition. However, in the Pt/Nb series, three devices with t_{Nb} < 4 nm show a positive DLT (negative FLT) sign.

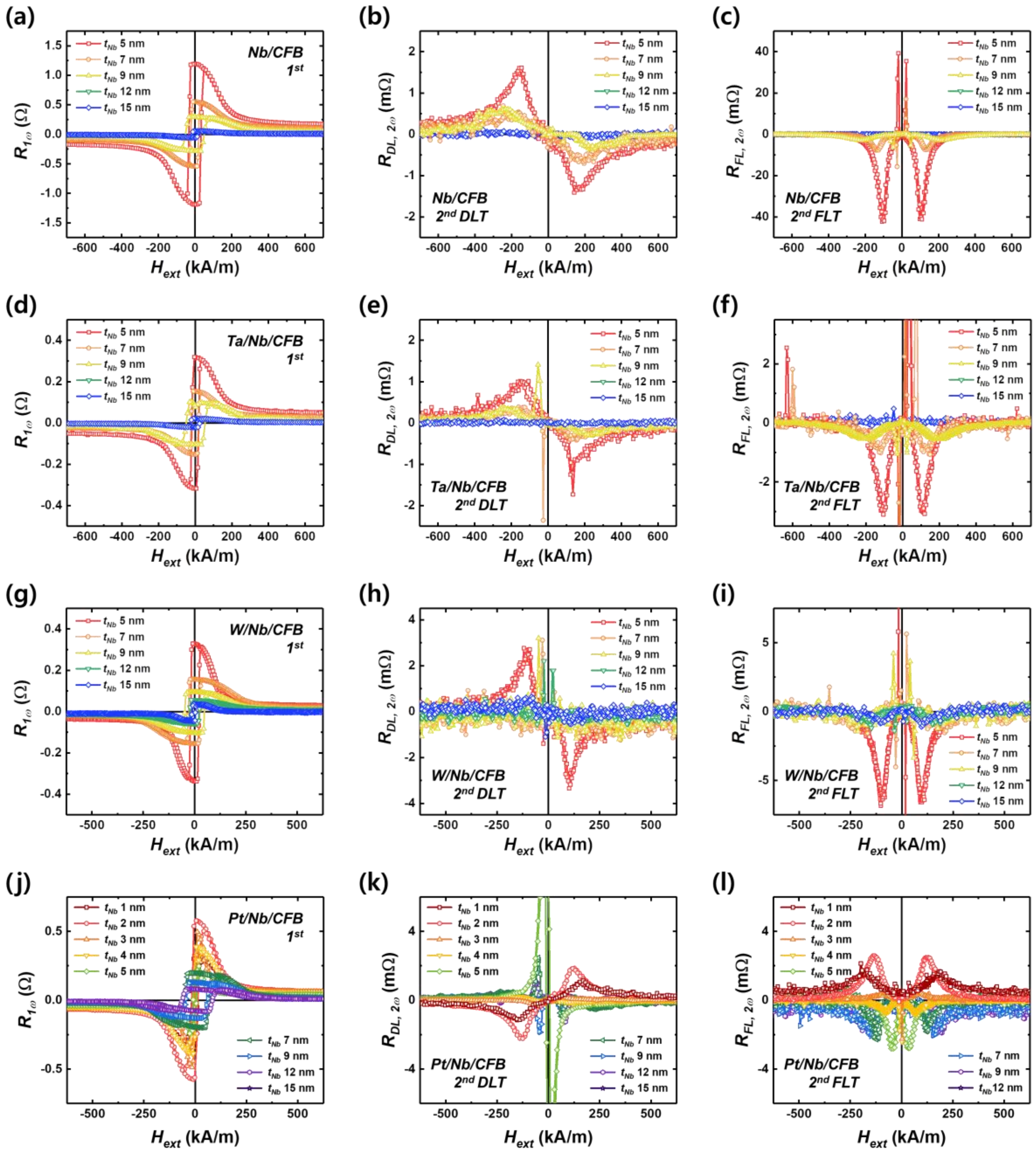

Figure S3. Raw harmonics data of the samples with PMA (a), (d), (g), and (j) are the first harmonics Hall resistance. (b), (e), (h), and (k) are the second damping-like Hall resistance, and (c), (f), (i), and (l) are the second field-like Hall resistance for all samples.

Section S4. Current-induced deterministic SOT switching

We utilized ion milling to create a dot-shaped structure in the CoFeB/MgO/Ta layers with a 5 μm diameter, enabling us to measure the SOT switching. In Figure S4, distinct SOT switching is evident in all Nb/CoFeB heterostructures. When an external magnetic field was applied along the +x axis, the ferromagnetic (FM) material exhibited a counterclockwise switching behavior. Conversely, when the external field was oriented along the -x axis, we observed a reversal in the switching polarity, compelling evidence of SOT-induced magnetization switching. In the Pt/Nb series, the magnetization rotated clockwise for films with t_{Nb} of 1 and 2 nm under the +200 Oe external field. However, magnetization rotated counterclockwise for films with t_{Nb} over 3 nm. The results show that for cases with a thin Nb, the spin current generated in the Pt layer dominates the current generated in the Nb or at the Nb/CoFeB interface and confirms the results from the harmonic measurement described in Figure 2 of the manuscript.

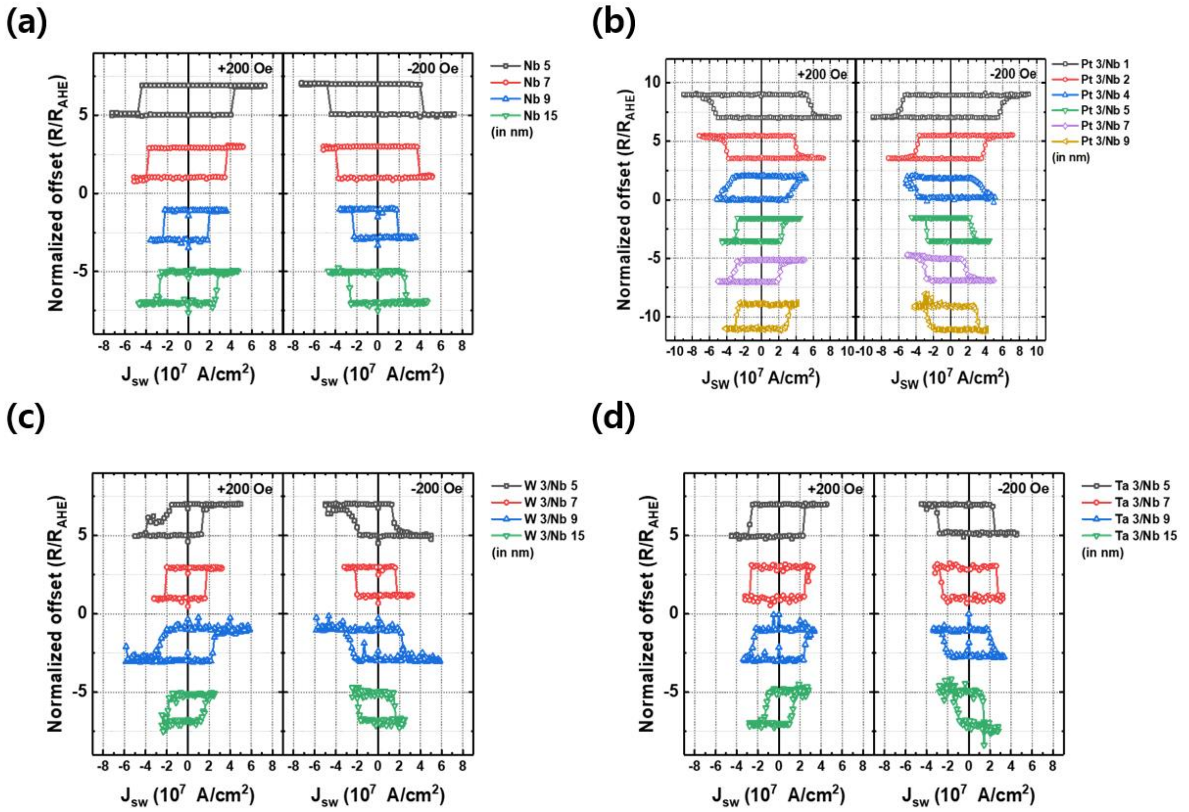

Figure S4. The normalized Hall resistance (*R*/*R*$_{AHE}$) as a function of current density (*J*) (a) Nb (t_{Nb})/CoFeB heterojunctions, (b) Pt 3/Nb (t_{Nb})/CoFeB heterojunctions, (c) W 3/Nb (t_{Nb})/CoFeB heterojunctions, (d) Ta 3/Nb (t_{Nb})/CoFeB heterojunctions.

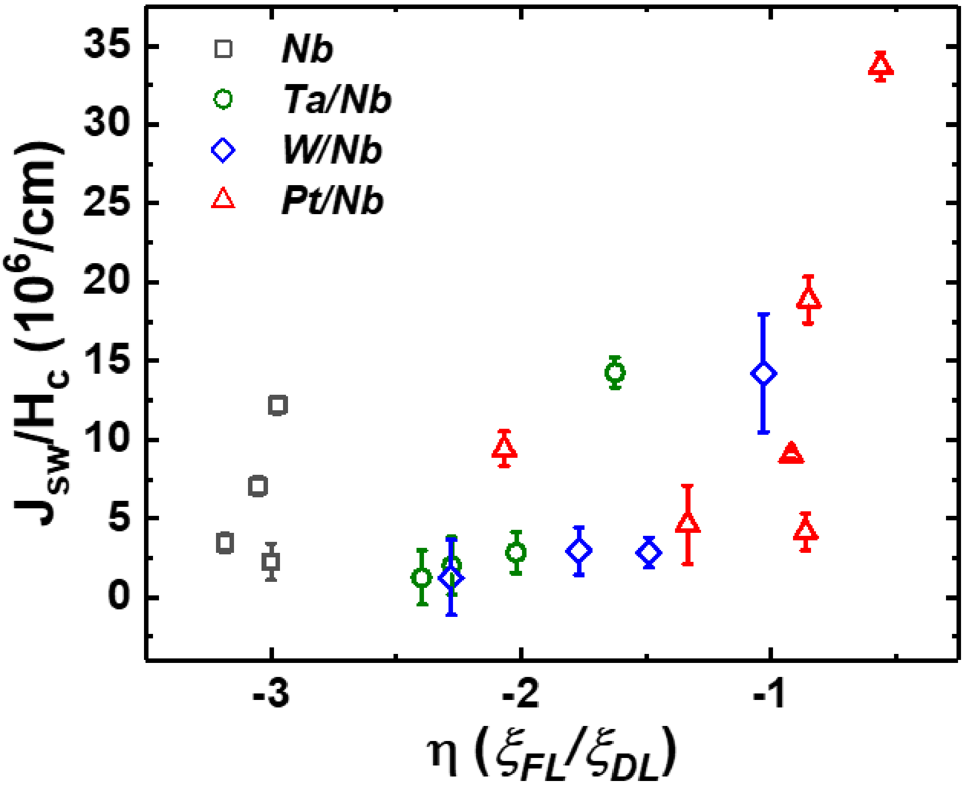

Figure S5. The switching current density (J_{sw}) normalized by coercive field (H_c) as a function of η. Each symbol with a different color and shape represents another series of Nb-based devices.

Section S5. The switching probability and the threshold current for the Nb-based SOT devices.

The electrical resistivity and current-induced SOT switching were measured using a 4-point probe station (MSTECH M7VC). We fabricated dot-shaped SOT devices using photolithography and ion milling. The milling stopped at the NM/FM interface due to the design of the dot-shaped structure consisting of a CoFeB/MgO/Ta layer with a 5 μm diameter. We applied current with a 10 μs pulse width and an external magnetic field parallel to the current direction during measurement. We induced a current without an external field to observe the switching probability and repeated the following four steps 50 times to obtain probability properties. Step (1): Applying the external field in +x (-x) direction. Step (2): Inducing current in the +x (-x) direction to first set the magnetization in the +z (-z) direction. Step (3): Turning off the external field. Step (4): Inducing current to settle magnetization along the y-axis. When we turned off the power supply of the electromagnet, the residual magnetic field applied to the device was 0.5 Oe or less. Switching was not secured when we conducted current-induced SOT switching under the residual magnetic field. Thus, we considered that the field had a negligible effect on the system. Steps (1) and (2) ensure independence between every switching trial. After the one cycle ends, the y-direction spins return to the magnetization easy-axis (±z). Each probability that spins rotate to the +z or -z-axis is 1/2, respectively, when the current amplitude exceeds the critical value defined as threshold current.

We experimentally defined the threshold current (I_{th}) as the switching probability is 49%. The switching measurement was conducted 50 times for each current, and the probability was represented in the blue dots. The area below the I_{th} is expressed in yellow, and the above region is green. The solid red line is the calculation results of the Boltzmann-sigmoid functions described as follows:

$$P_{sw} = 0.5(1 - \frac{1}{1 + e^{\left(\frac{x - x_0}{dx}\right)}}) \quad \text{(S13)}$$

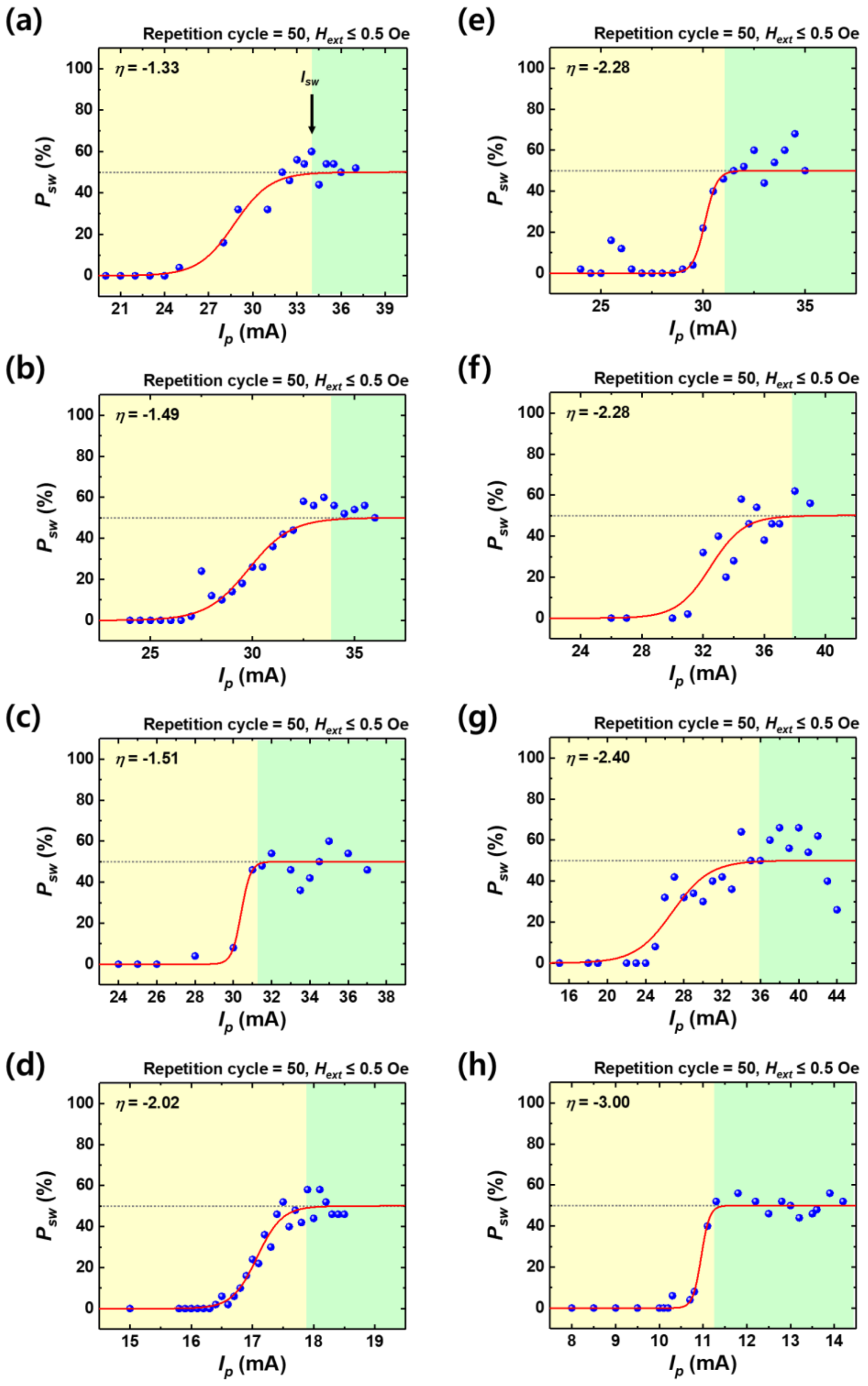

Figure S6. The switching probability distribution for the Nb-based SOT devices While we inject the current pulse, the magnetization lies in the in-plane direction. When stopping the current supply, it aligns randomly in the +z or -z direction. The aligning direction was characterized by reading the transverse Hall voltage. The probability of switching under the condition that 50 write operations are performed for each current level. The Red line is a fitting curve using the Boltzmann sigmoidal function. We experimentally defined the I_{th} as the x value that makes P_{sw} = 0.49.

Section S6. The threshold current and current density as a function of η.

We measured the switching probability by injecting the current and extracted the threshold current (I_{th}) as the switching polarity is 49% for the Nb-based SOT device. To determine the effect of FLT on SOT switching, we plot the I_{th} as a function of η. I_{th} does not tend to change in η. The threshold current density (J_{th}) reflecting the dimension of each device was also plotted as varying η. J_{th} shows a weak tendency that as the η increases, J_{th} also increases.

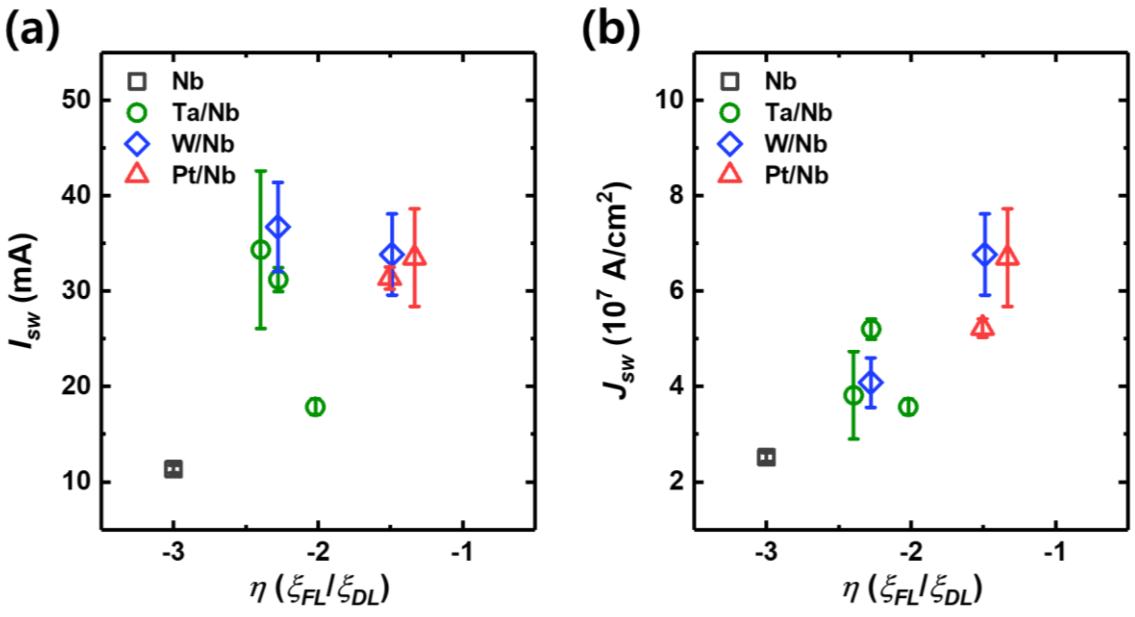

Figure S7. The switching current and current density as a function of η (a) Switching current (I_{sw}). (b) switching current density (J_{sw}) as a function of η for the Nb-SOT device. Each symbol with a different shape and color depicts another series of Nb-based devices.

Section S7. Coercive field and anisotropy energy of Nb-based films

We calculated the coercive field (H_c) and effective anisotropy energy (K_{eff}) of Nb-based films and analyzed the effects of the parameters on SOT switching. The calculation was made using the M-H curves in Section S2 in Supporting Information.

The H_c of stacks with t_{Nb} of less than 7 nm exhibited a limited value of ~ 0.2 kA/m. Above 7 nm, H_c becomes larger and saturated, and the level is significant at ~1.4 kA/m in the W/Nb and Ta/Nb series and relatively small at ~0.9 kA/m in the Nb and Pt/Nb series. The K_{eff} does not tend to the thickness of the Nb layer. Still, all the series exhibited a value of about 1 Merg/cm^3, which means all films showed well-defined perpendicular magnetic anisotropy.

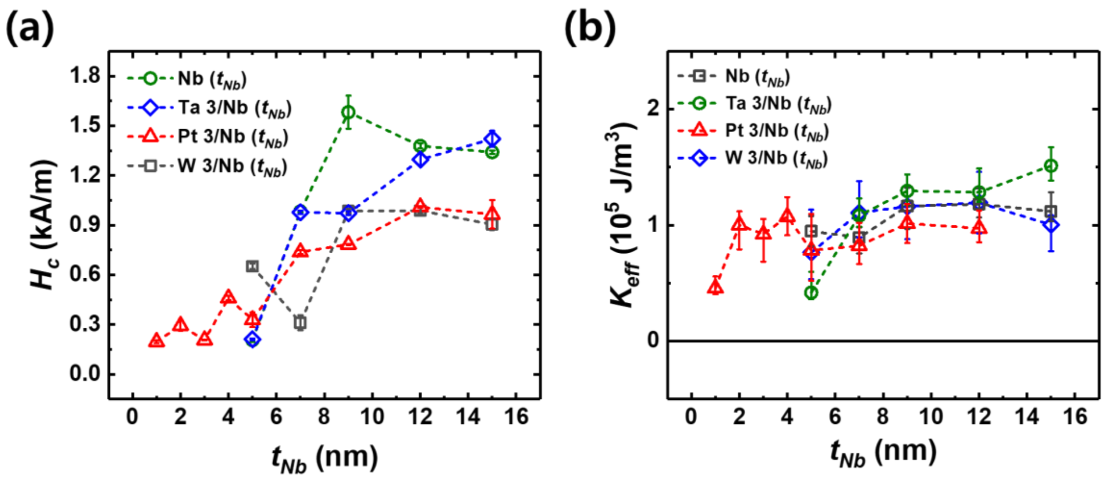

Figure S8. H_c and K_{eff} as a function of t_{Nb}. (a) H_c and (b) K_{eff} according to t_{Nb} for all the samples for Nb-based SOT devices. Points have different colors and shapes and mean Nb-based files of other series.

Section S8. Random bit generation using Nb-based trilayer devices

We used three SOT devices with different eta values; device #1: device #1: Nb 9/CoFeB 1 nm (η = -3.03), device #2: W 3/Nb 15/CoFeB 1 nm (η = -2.18), and device #3: Pt 3/Nb 9/CoFeB 1.1 nm (η = -1.53).

Figure S9a shows the sequence of the random number generation experiment using device #1. For the first step, we induced deterministic SOT switching by applying an external field along the current injection direction. Then, we turned off the external field and applied a 10 μs I_p large enough to arrange the magnetization to the in-plane direction completely. We repeatedly inject an I_p larger than the I_{sw} of each device. After one write operation, we specified one bit by reading the Hall resistance (R_H). After every 1,000 times operations, we conducted the deterministic SOT switching with an external magnetic field to confirm the reliability of random bits created from a working SOT device.

If the write operation is performed several times, thermal degradation of the device occurs. The repeated pulse injections leading to temperature elevation and induced damage result in the degradation of the magnetic anisotropy of the device, thereby influencing the operational range (R_H). Hence, the variability of device resistance, which represents the operational range, fluctuates with each successive measurement iteration. The impact of this deterioration was mitigated by employing the R_H values obtained from deterministic switching measurements conducted before and after every 1000 write operations. In the event of damage to the device leading to the absence of deterministic switching, the preceding 1000 data sets are discarded.

The number of bits generated using SOT is as follows for each device: device #1 (η = -3.03): 308,683 bits, device #2 (η = -2.18): 188,406 bits, and device #3 (η = -1.53): 392,156 bits.

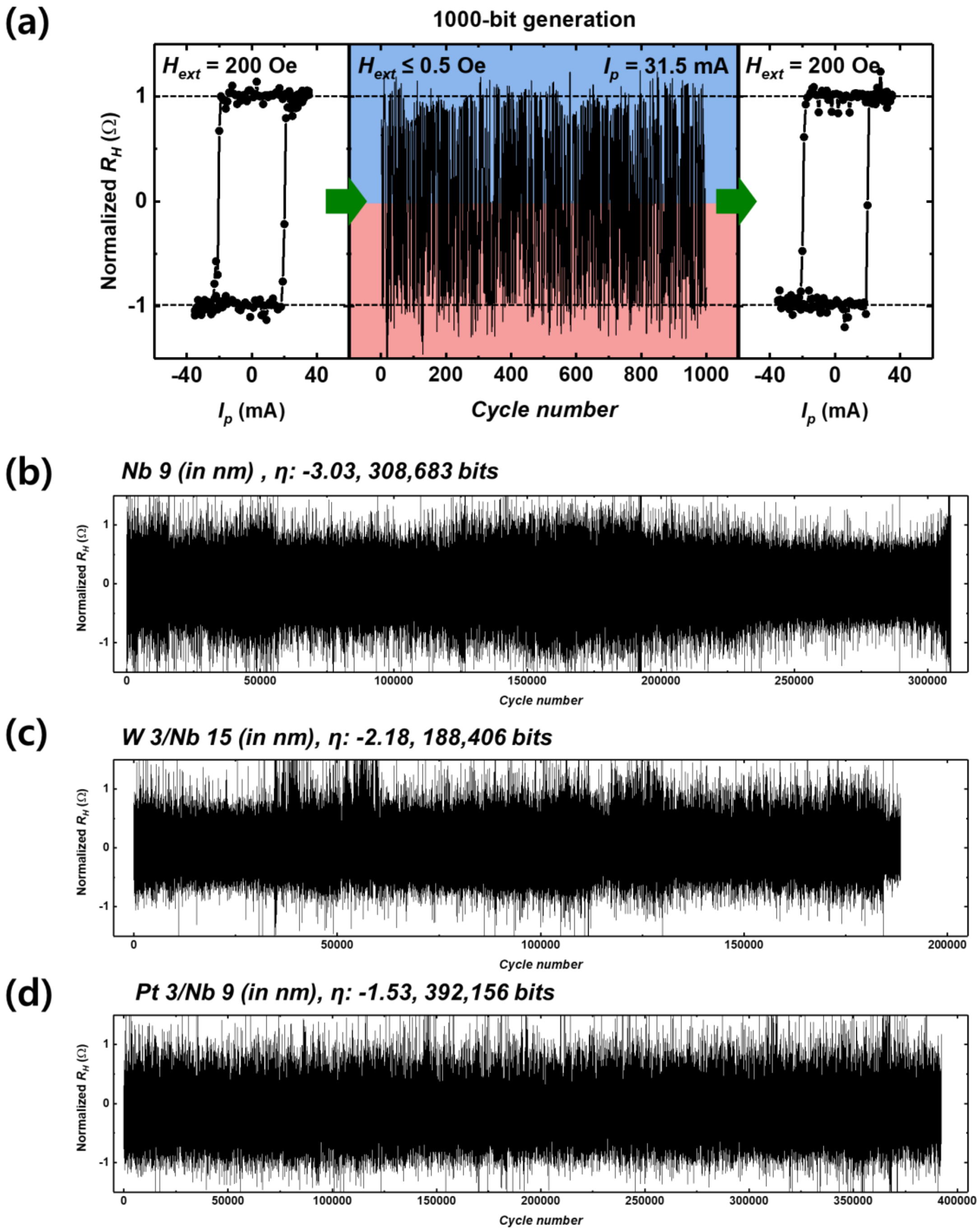

Figure S9. Normalized R_H for random number bits (a) A sequence of random number generation using a trilayer device. First, we assured deterministic-SOT switching with an external magnetic field of 200 Oe. After that, the external magnetic field was turned off, and we injected a pulse of 31.5 mA with a length of 10 us (the pulse amplitude is different for each device) 1000 times and measured the Hall voltage after each injection. After 1000 write operations, the post-switching measurement was performed to check the state of the device to confirm the validity of the random string. Random bits generated from (b) device #1: Nb 9/CoFeB 1 nm (η = -3.03), (c) device #2: W 3/Nb 15/CoFeB 1 nm (η = -2.18), and (d) device #3: Pt 3/Nb 9/CoFeB 1.1 nm (η = -1.53).

Section S9. Threshold current density dependence on pulse width and damping parameter

Figure S10 shows Eqs. (3-5) in the manuscript compared to simulations with α = 0.02 and pulse width = 50 ns in Figure S10a, α = 0.02 and pulse width = 5 ns in Figure S8b, and α = 0.2 and pulse width = 50 ns in Figure S10c. Meanwhile, the simulations in Figure S10a fit with Eqs. (3-5), the simulations in Figure S10b with $\eta \leq 2$ and Fig. S10c with $\eta \leq 0$ do not work with Eqs. (S14-S16). Since the SOT switching with $\eta \leq 0$ is induced by the anti-dumping mechanism, a longer pulse width and larger damping parameter result in a larger threshold current density. These results show that SOT devices with η > 1 have high-speed and low-power consumption characteristics that can be utilized for TRNG.

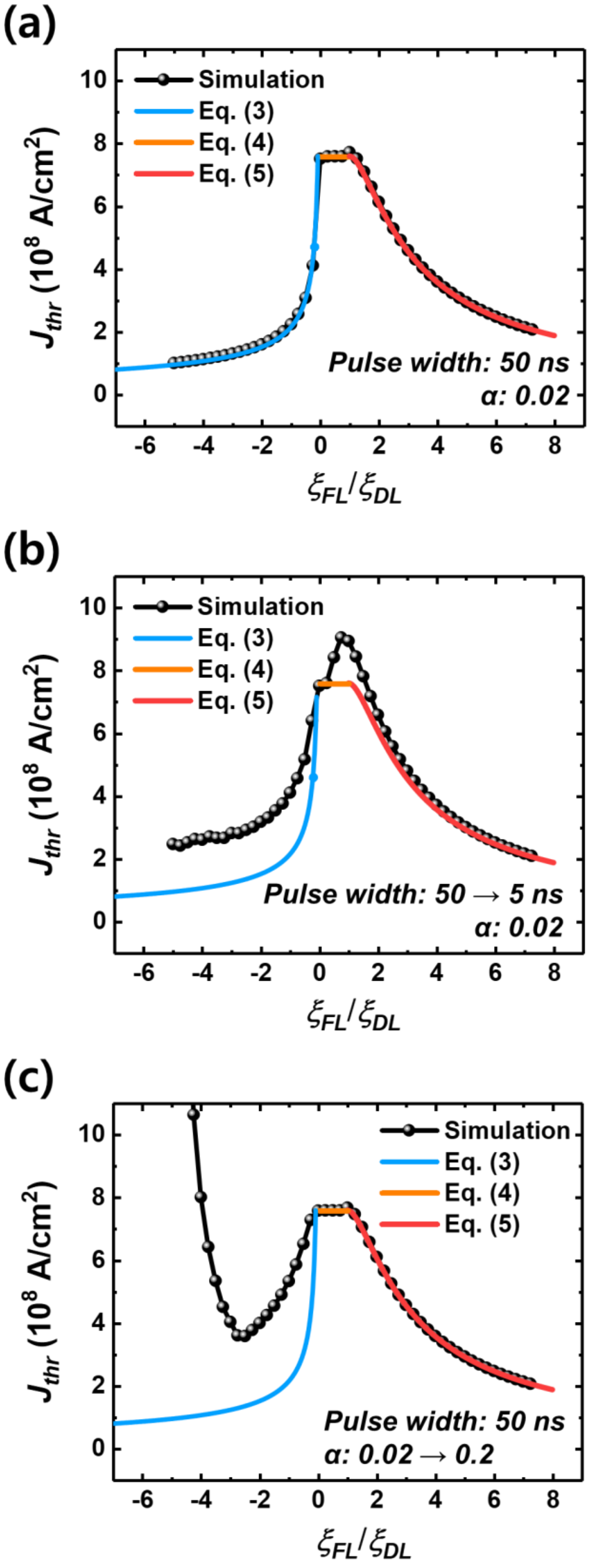

Figure S10. Eqs. (3-5) compared to simulations with (a) α = 0.02 and pulse width = 50 ns, (b) α = 0.02 and pulse width = 5 ns, and (c) α = 0.2 and pulse width = 50 ns. Other parameters for simulations are: $H_k = 2000\ \mathrm{A/m}, \alpha = 0.02, \theta_D = -0.04, 0 \leq J \leq 3 \times 10^8 \mathrm{A/cm^2}, M_s = 1000\ \mathrm{kA/m}, t_f = 1$ nm.

References

S1. W. F. Brown, Jr., *Phys. Rev*. **130**, 1677 (1963).

S2. W. F. Brown, Jr., *IEEE Trans. Magn*. **15**, 1196 (1979).

S3. William T. Coffey and Yuri P. Kalmykov, Thermal fluctuations of magnetic nanoparticles: Fifty years after Brown, *J. Appl. Phys.* **112**, 121301 (2012).

S4. W. H. Butler, T. Mewes, C. K. A. Mewes, P. B. Visscher, W. H. Rippard, S. E. Russek and R. Heindl, Switching Distributions for Perpendicular Spin-Torque Devices Within the Macrospin Approximation, *IEEE Trans. Magn.* **48**, 12 (2012).

S5. D. Das, and X. Fong, A Fokker–Planck Approach for Modeling the Stochastic Phenomena in Magnetic and Resistive Random Access Memory Devices, *IEEE Trans. Electron. Devices* **68**, 12 6124-6131 (2021).

S6. M. Hayashi, J. Kim, M. Yamanouchi, and H. Ohno, Quantitative characterization of the spin-orbit torque using harmonic Hall voltage measurements, *Phys. Rev. B* **89**, 144425 (2014).

S7. T. Taniguchi, S. Mitani, and M. Hayashi, Critical current destabilizing perpendicular magnetization by the spin Hall effect, *Phys. Rev. B* **92**, 024428 (2015).

S8. D. Zhu, and W. Zhao, Threshold Current Density for Perpendicular Magnetization Switching Through Spin-Orbit Torque, *Phys. Rev. Appl.* **13**, 044078 (2020).
